# Descriptive Temporal Epidemiology of Tularemia Using Case Reports and Hospitalization Data in the United States, 2000–2022

**DOI:** 10.3390/pathogens15010086

**Published:** 2026-01-13

**Authors:** Chad L. Cross, Bryson Carrier, Louisa A. Messenger

**Affiliations:** 1 School of Veterinary Medicine, Texas Tech University, Amarillo, TX 79106, USA; 2 UNLV Parasitology & Vector Biology (PARAVEC) Laboratory, School of Public Health, University of Nevada, Las Vegas, NV 89154, USA; 3 TTTSVM Parasitology & Vector Biology (PARAVEC) Laboratory, School of Veterinary Medicine, Texas Tech University, Amarillo, TX 79106, USA; 4 School of Kinesiology, University of Michigan, Ann Arbor, MI 48109, USA; bryson.carrier@oit.edu; 5 Department of Natural Sciences, Oregon Institute of Technology, Klamath Fall, OR 97601, USA; 6 Department of Environmental & Global Health, School of Public Health, University of Nevada, Las Vegas, NV 89154, USA; louisa.messenger@unlv.edu

**Keywords:** tularemia, epidemiological surveillance, rare diseases, healthcare utilization, zoonotic diseases, indirect surveillance

## Abstract

Tularemia is a well-known zoonotic disease around the world, with particularly high rates in certain geographic areas of the U.S. Though the disease is regularly reported, it is classified as a rare condition owing to the relatively low number of cases detected annually. Interestingly, however, the number of cases in the U.S. has shown a positive upward trend through time. The aim of this study was to summarize, interpret, compare, and contextualize temporal trends in tularemia epidemiology at the national scale within the U.S. utilizing long-term data sets encompassing the 23-year span from 2000 to 2022. We used two secondary data sets: (1) case data reports from the National Notifiable Disease Surveillance System (NNDSS) of the Centers for Disease Control and Prevention (CDC) and (2) the National Inpatient Sample (NIS) of hospitalization discharge records. In addition to investigating patterns, we were interested in the utility of using hospital discharge records as a means of indirect epidemiological surveillance of this rare disease. Both data sets highlight the high variability in annual cases through time but underscore the highest risk of disease among patients classified as White and male, as well as the extraordinarily high rates among American Indian/Alaska Native populations, particularly those with pulmonary tularemia disease. Descriptive epidemiological summaries and statistical comparisons are provided across the time series for sex, age, ethnoracial identity, and geography; hospitalization characteristics are also described. Our desire to use case rates from hospitalization records as a surrogate for CDC case incidence rates did not provide the desired precision, though hospital discharge records do provide valuable and useful information necessary to estimate general high-risk groups for tularemia through time.

## 1. Introduction

Tularemia is a well-known zoonotic disease resulting from infection with the pathogenic Gram-negative coccobacillus *Francisella tularensis*, of which two distinct variants are found in the U.S. (Type A and Type B) [1]. There are several colloquial names given to tularemia, including “rabbit fever” and “deer fly fever,” the former of which resulted from historic commonality of the pathogen among rabbit hunters and the latter in reference to the arthropod deer fly (Tabanidae: *Chrysops* spp.) vector; however, ticks (Ixodidae: *Dermacentor variabilis*, *Dermacentor andersoni*, and *Amblyomma americanum*) are also well-known vectors. Tularemia, however, does not need to be vectored to pose a significant threat. Historical accounts of relatively low-dose exposure to the aerosolized pathogen are well documented [2], hence resulting in its addition to the U.S. National Institute of Allergy and Infectious Diseases biodefense pathogen list [3].

Though tularemia is considered relatively rare in the U.S., Rich et al. provide results suggesting that not only are cases increasing, but that there has been a startling increase of 56% over two decades, with rates rising from 0.041 per 100 K in 2001–2010 to 0.064 per 100 K from 2011–2022 [4]. Importantly as well, these authors highlight relative differences among groups, with particularly high rates of disease occurring in American Indian/Alaskan Natives and among children 5–9 years of age. Though most cases in the U.S. have been reported in Oklahoma, Arkansas, Kansas, and Missouri, large increases in both human and animal cases in other states (e.g., Minnesota) have prompted health officials to take a closer look at the current status of this disease [4,5].

Tularemia treatment and appropriate clinical care rely heavily on clinical judgment surrounding the specific circumstances of the patient (e.g., infection with Type A/B subspecies variants, severity of disease, and patient age), but specific antimicrobial therapies are still the traditional hallmarks of successful care [6]. Vaccination in the U.S. for tularemia is not widely available outside of the military and some high-probability exposure laboratory personnel, though the Live Vaccine Strain (LVS) has been shown to produce cell-mediate immunological response for at least three decades [2,7].

Outcomes from hospitalization of tularemia patients are not well documented in the recent literature, though Widerström and colleagues were able to observe 67 Swedish patients with severe respiratory tularemia. Among the relatively older population (median age 66 years) of mostly male patients (81% males), mortality rates were low (1 out of 67) when treated with fluoroquinolones [8]. Because little information is reported concerning tularemia in the inpatient setting in the U.S., filling this information gap and analyzing available data on the characteristics of hospitalized patients may prove valuable in understanding disease etiology and outcome as well as providing information on disease trends.

The aim of this study was to summarize, interpret, compare, and contextualize temporal trends in tularemia epidemiology at the national scale within the U.S. utilizing two long-term secondary data sets encompassing the 23-year span from 2000 to 2022. We sought to utilize the longitudinal summary studies published by the CDC, characterizing cases from 2001 to 2010 [9] and 2011 to 2022 [4], by examining an expansion of this time series across a continuous 23-year span, and then to provide a comparison with patients who had been hospitalized during the same period and who had a tularemia diagnosis in their diagnostic record. Though the goal of the study was to provide a comprehensive understanding and contextualization of epidemiological disease rates through a long span of time, an important underlying objective of the analysis was to investigate the utility of long-term secondary hospitalization data sets for indirect disease surveillance and as a potential surrogate for understanding patterns in diseases.

## 2. Materials and Methods

This study provides a descriptive epidemiological picture of tularemia from two perspectives—namely, (1) reported cases to the CDC and (2) U.S. hospitalizations in which tularemia is a listed diagnosis of a patient record. The first data set represented cases from the National Notifiable Disease Surveillance System (NNDSS) collated manually from annual reports prior to 2015 and from more recent electronic sources from the CDC WONDER database [10,11]. The second data set represented inpatient hospital discharge records maintained by the Healthcare Utilization Project (HCUP) of the Agency for Healthcare Research and Quality (AHRQ). These data serve as the nation’s largest available inpatient sample, the National/Nationwide Inpatient Sample (NIS), which is collated from state inpatient databases. The entire annual sample of discharge records is a weighted, 20% stratified sample from participating community hospitals across the U.S. [12,13]. The data, when weighted properly for analysis, represent approximately 35–40 million hospital discharges annually and can provide unbiased national estimates [14].

The NIS contains many diagnostic codes for each discharge record (15–40 codes depending on the year) included in the annual weighted sample, making each year of data several Gb in size. When expanded across the 23 years of data utilized in this study, the full database includes over 850 million discharge records, making analysis challenging simply owing to file size. Therefore, it was necessary to first extract only the relevant tularemia records as an initial step in the analysis. Following a previously published and verified brute-force algorithm [15], we utilized the International Classification of Disease (ICD) codes for tularemia to extract the records of interest. Note that two versions of ICD codes were used because our temporal data set straddled the fourth quarter of 2015 when the U.S. switched from ICD version 9 to ICD version 10 (Table 1).

Following data extraction and compilation, tularemia counts were compiled by year for both the NIS and NNDSS data sets. Because epidemiological rates are most useful compared to raw case counts as they include the population at risk at a given time in the calculation and hence are decidedly more useful for direct comparisons, we generally focus on reporting rates per 100,000 (100 K) in the results. The denominator in these used the U.S. Census Bureau population data from 2017 [16], which was chosen to match the denominator population used in the recently published CDC 2025 MMWR tularemia estimates [4]. We note that NNDSS data are known new or probable cases, and hence represent incidence rates, but that NIS data technically represent period prevalence rates (i.e., cases reported over a one-year period). However, because the probability of any one tularemia case being randomly chosen in the weighted NIS data set in multiple years is considered extremely small, period prevalence in the NIS data set is a close approximation to an incident rate and will be referred to as such herein.

The intent of this study was to investigate the utility of using inpatient hospitalization records both to understand temporal trends and to discern if this indirect epidemiological surveillance method using NIS might prove useful as a surrogate for understanding probable or confirmed disease trends collected and summarized in the NNDSS. We characterized the analyses and results using several available variables in each of the data sets as provided. It is noted that these categorizations were used exactly as reported within the standardized databases (e.g., sex was reported in the data as “male” and “female”; ethnoracial categories were also prescribed in the secondary data and are reported as provided).

We first report an overview of general temporal trends. These trends are summarized as both case counts for completeness and as rates as an epidemiological result. Trend lines through the 23 years of data were estimated using autoregressive models to account for temporal autocorrelation. Additionally, monotonic trends were tested using the Mann–Kendall test for each data set to investigate potential generalized linear directionality (i.e., increasing, decreasing, or stable). Potential associations between rates for NIS and NNDSS data were examined using Pearson correlation. Potential differences between sexes were tested with an independent-samples *t*-test. Age groups and ethnoracial categories were compared using an ANOVA for global comparisons followed by Bonferroni-adjusted post hoc comparisons if the global test was significant. For total hospitalization charges and length of stay in the NIS data, descriptive statistics (e.g., mean and SD) are provided using case counts as a weighting variable across years. Additionally, yearly total hospitalization charges were adjusted annually to 2024 U.S. dollars using the Consumer Price Index [17] prior to the calculation of descriptive statistics; this provided a way to equilibrate the charges for inflation using the most recent full year of inflation data (i.e., 2024). Geographic trends could not be fully explicated and statistically compared for both data sets owing to collation of data into different regional representations, which are standardized within, but not between, each data set; further, we did not have access to regional data for NIS until 2018. Therefore, we provide geographic visualizations using mapping software and discuss overall observations. For all statistical tests when available, we report *p*-values based on 10,000 Monte Carlo bootstrapped resamples instead of relying on asymptotic distributional estimates. All statistical analyses and data visualizations were developed through syntax in SAS software (v. 9.4; SAS, Cary, NC, USA).

## 3. Results

### 3.1. Temporal Trends and Disease Categorization

**Figure 1:**
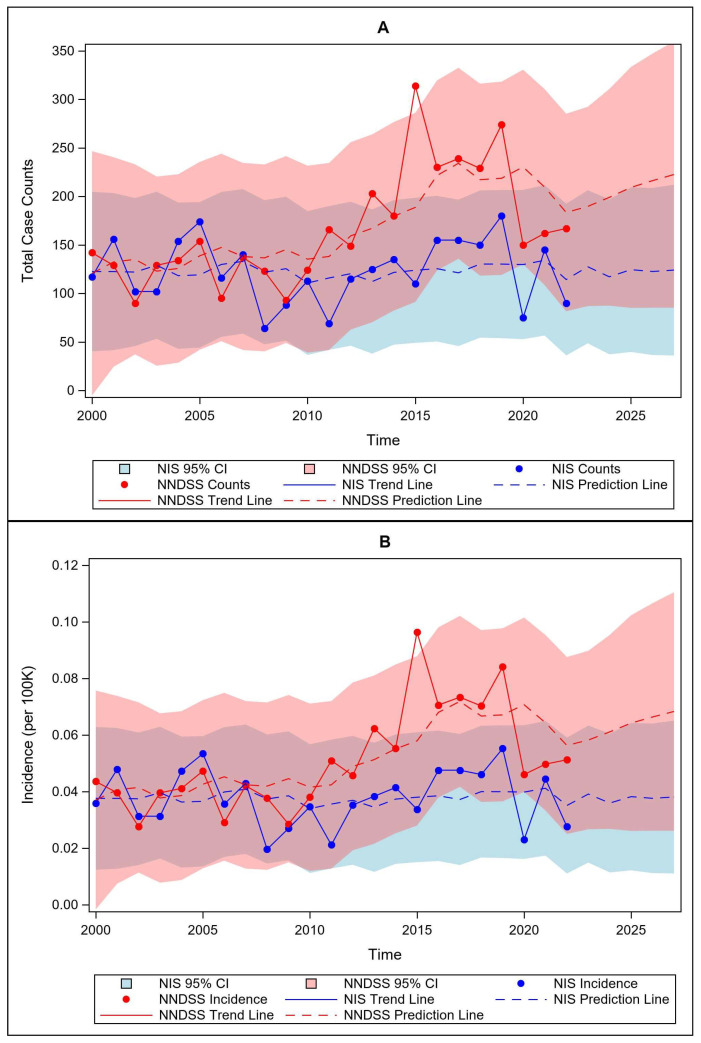
Temporal trends in (**A**) tularemia total case counts and (**B**) incidence per 100,000 population through time. Both figures display point estimates with connected trend lines, confidence intervals, and autoregressive prediction lines. Note that overlap of confidence intervals is shown as the purple region in the graph.

The total number of NNDSS annual cases ranged from 90 to 314 (median = 150), with incidence rates ranging from 0.03 to 0.10 per 100 K (median = 0.05). Hospitalization discharge records indicated a range of 64–180 cases (median = 117) and rates ranging from 0.02 to 0.06 per 100 K (median = 0.04). There was a great deal of temporal variability in cases and rates, with demonstrable differences between NIS and NNDSS cases after 2010. In fact, inpatient records suggested no temporal trend (MK = 0.04, *p* = 0.3977), whereas NNDSS records demonstrated a significant increasing trend through time (MK = 0.48, *p* = 0.0006) (Figure 1A,B).

Autoregression models for lags between AR1 and AR3 were tested for each prediction model, with higher lag times not required after visualizing autocorrelation plots. For both NIS and NNDSS data, autoregressive timelags of AR2 provided optimal fit and minimized the Akaike Information Criteria (AIC) while providing acceptable autocorrelation measures (Durbin–Watson statistics between 1 and 2). These autoregressive trend lines were plotted along with case counts and incidence rates per 100 K and carried forward three additional years (i.e., through 2025), demonstrating predicted continued increases in NNDSS tularemia through time but relatively stable hospitalizations (Figure 1). Interestingly, however, there is a significant correlation between the actual rates between the two data sets (r = 0.39, *p* = 0.0351), and though the effect size is only small-to-moderate, an NNDSS incidence rate can be estimated for a given year using the NIS rate (i.e., NNDSS rate = 0.025 + 0.692 × (NIS rate)), and similarly, expected hospitalization burden could be estimated knowing the NNDSS rate (NIS rate = 0.026 + 0.223 × (NNDSS rate)), though the estimates would likely lack reliability.

Use of hospitalization data along with ICD coding provides much more granular information about individual discharge records than can be found in publicly available NNDSS data, particularly relative to diagnostic subcategories of tularemia disease (Table 2). Generalized or unspecified tularemia are most often the diagnostic categories reported, but interesting associations can be found that are informative for hospital care and understanding particular risks for patient group variables. For example, ulceroglandular and oculoglandular tularemia are highly prevalent in those <5 years and among American Indians/Alaska Natives, who represent the highest incidents rates annually; pulmonary tularemia is demonstrably the most problematic (Table 2).

### 3.2. Trends and Comparisons by Sex

**Figure 2:**
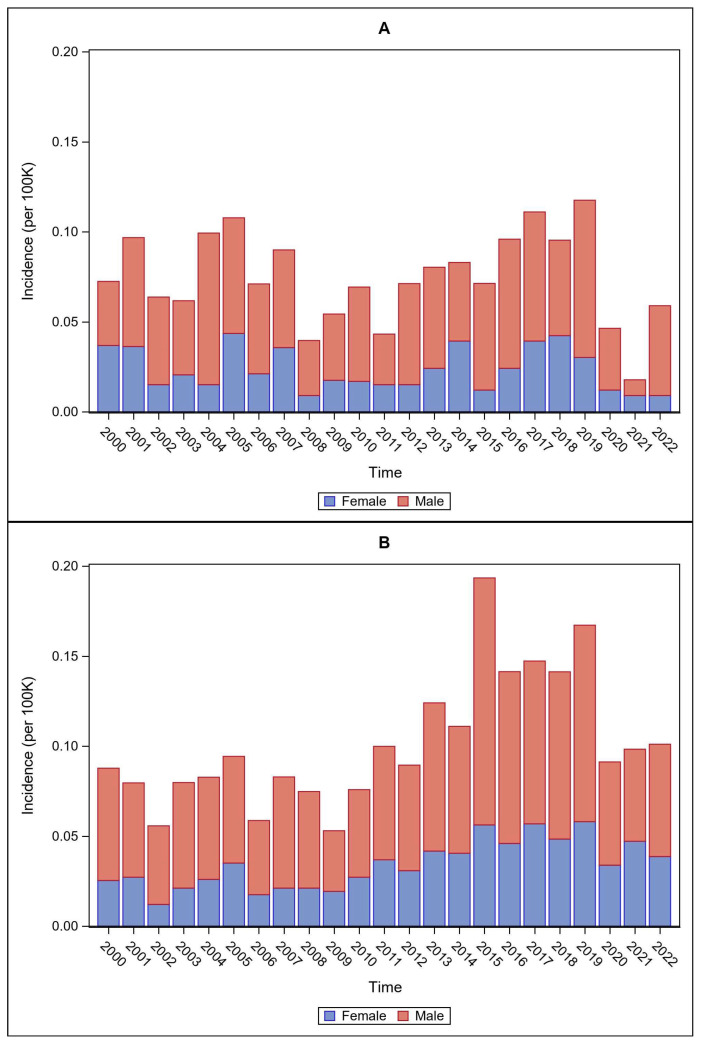
Temporal trends in tularemia incidence per 100,000 population by sex classification for (**A**) NIS and (**B**) NNDSS data.

Sex differences in mean incidence rates across time were significant for NIS data, with males higher than females (t = 6.31, *p* < 0.0001; males: mean = 0.05, SD = 0.018; females: mean = 0.02, SD = 0.002) (Figure 2A). This difference was also true for NNDSS data (t = 5.71, *p* < 0.0001), with males (mean = 0.07, SD = 0.02) significantly higher than females (mean = 0.03, SD = 0.013) (Figure 2B).

### 3.3. Trends and Comparisons by Age

**Figure 3:**
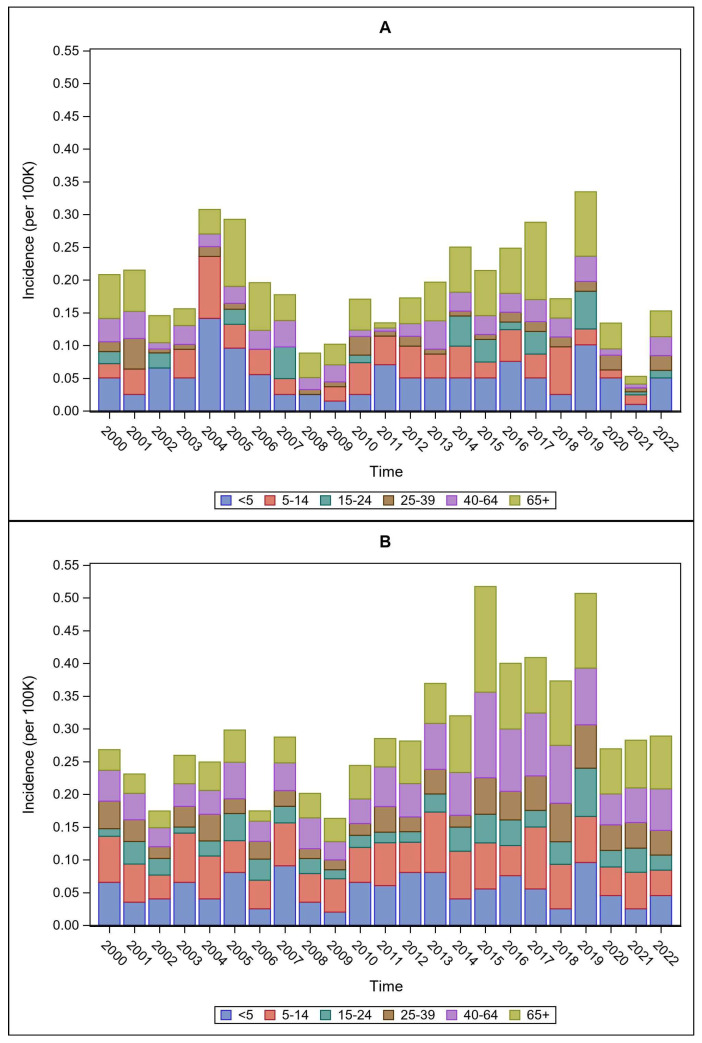
Temporal trends in tularemia incidence per 100,000 population by age group classification for (**A**) NIS and (**B**) NNDSS data.

Mean case incidence rates were not equitable across ages for the NIS data (F = 15.82, *p* < 0.0001) (Figure 3A), with the lowest rates found in the 25–39 age group (mean = 0.01, SD = 0.010) and the highest mean rates in the 65+ age group (mean = 0.05, SD = 0.028). Significant corrected post hoc comparisons (*p* < 0.05) indicated that (1) those <5 years had significantly higher rates compared to all groups except 5–14 and 65+; (2) those 5–14 years had higher rates than 15–24 and 25–39; and (3) those 15–24, 25–39, and 40–64 years had lower rates compared to 65+.

Similarly, mean case incidence rates were not equal across ages for the NNDSS data (F = 9.45, *p* < 0.0001) (Figure 3B), with the lowest rates found in the 15–24 age group (mean = 0.03, SD = 0.015) and the highest mean rates in the 65+ age group (mean = 0.06, SD = 0.034). Significant corrected post hoc comparisons (*p* < 0.05) indicated that (1) those <5 years had significantly lower rates than 15–24; (2) those 5–14 years had significantly higher rates than those 15–24 and 25–39; (3) those 15–24 years had significantly lower rates compared to those 40–64 and 65+; and (4) those 25–39 years had significantly lower rates than those 40–64.

### 3.4. Trends and Comparisons by Ethnoracial Identity

**Figure 4:**
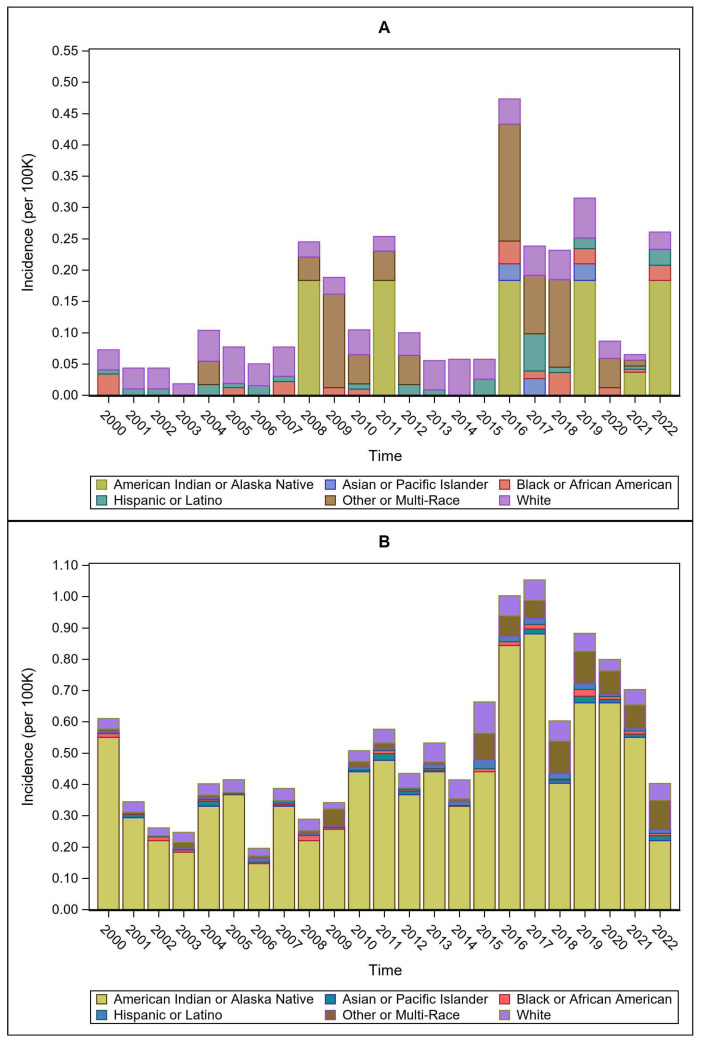
Temporal trends in tularemia incidence per 100,000 population by ethnoracial identity classification for (**A**) NIS and (**B**) NNDSS data.

Mean incidence rates showed significant differences across ethnoracial identity groups in the NIS data (F = 4.08, *p* = 0.0018) (Figure 4A), with the lowest mean rates found in the Asian/Pacific Islander group (mean = 0.003, SD = 0.009) and the highest mean rates in the American Indian/Alaska Native group (mean = 0.04, SD = 0.077). Significant post hoc comparisons indicated that the Asian/Pacific Islander group had lower rates than both the White group (*p* = 0.0442) and the American Indian/Alaska Native group (*p* = 0.0139).

Mean incidence rates were similarly different across ethnoracial identity groups in the NNDSS data (F = 88.38, *p* < 0.0001) (Figure 4B), with the lowest mean rates found in the Asian/Pacific Islander group (mean = 0.01, SD = 0.005) and the highest found in the American Indian/Alaska Native group (mean = 0.42, SD = 0.199). Significant post hoc comparisons confirmed the very high rates for the American Indian/Alaska Native group, as it was significantly higher than all other groups (*p* < 0.0001). The Asian/Pacific Islander group was also significantly lower than the other group (*p* < 0.0001).

### 3.5. Hospitalization Summary

The NIS inpatient data provide granular information useful for understanding characteristics of the hospitalized population. The overall median total cost for a record that contained a tularemia diagnosis was USD 63,953 (mean = USD 72,564, 95% CI: USD 48,609–USD 96,520) in 2024 dollars based on a median stay of 6.8 days (mean = 6.9 days, 95% CI: 6.0–7.7 days). Because a tularemia diagnosis may be one of many diagnoses in a given patient discharge record, total costs and length of stay are linked to potential comorbidities that also are diagnosed, or that may be a principal reason for the visit. For example, sudden fever of unknown origin, fatigue, dyspnea, etc., may lead to follow-up laboratory testing for a causative agent, in which case a tularemia diagnosis may be verified. Of those records with a tularemia diagnosis, the most common comorbid treatment conditions were related to heart disease (27.4%), hyposmolality or electrolyte imbalance (10.9%), respiratory failure (9.8%), kidney disease (6.1%), and anemia (4.8%).

### 3.6. Geographic Summary

**Figure 5:**
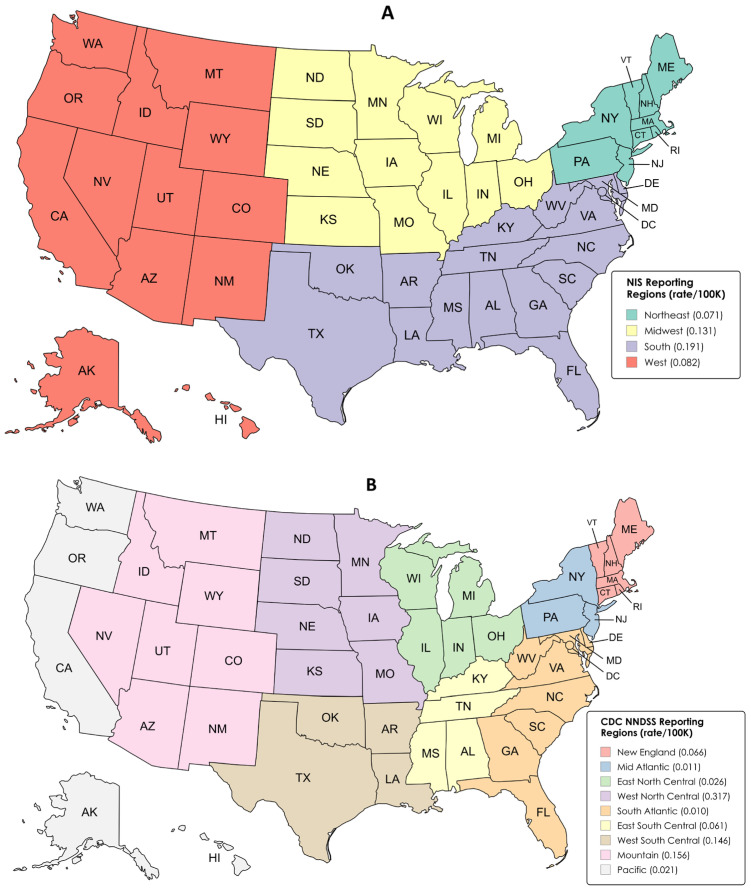
Spatial representation of rates in tularemia incidence per 100,000 population by reporting region for (**A**) NIS (2018–2022) and (**B**) NNDSS (2000–2022) data. Regional rates are shown by color, with rate/100 K provided in the map key. Maps were created using online MapChart software (https://www.mapchart.net/index.html (accessed on 18 November 2025).

Geographic regions are defined differently in the NNDSS and NIS data; hence, direct spatial comparisons are not possible. However, general spatial trends are notable. NIS tularemia discharges suggest that hospitalization rates are highest in the Midwest and South and lowest in the Northeastern U.S. (Figure 5A). Unfortunately, regional geographic information was only available in the NIS database from 2018 to 2022. Verified CDC NNDSS case rates are demonstrably highest among the states in the Central U.S. (West North Central Region, Mountain Region, and West South Central Region) and lowest along the coasts (Figure 5B).

## 4. Discussion

Though tularemia is classified as a rare disease [4,18], presence of the causative pathogen is not uncommon in the environment, with potentially severe consequences for human and animal health. In fact, it is estimated to be the pathogen with the broadest list of suspected wild and domestic susceptible hosts (>250 species) [19,20], so the relatively small number of reported human cases may appear lower than might otherwise be expected. This is perhaps due to underestimation of cases that go unreported owing to mild symptoms, uncertainty about diagnosis resulting from lack of experience or knowledge about the pathogen, and potentially use of antibiotic treatments for febrile cases of unknown origin that do not get a confirmed laboratory diagnosis [21]. Regardless of its classification as a rare disease, tularemia cases occur consistently, and quite variably, across the U.S. each year (Figure 1).

Epidemiological surveillance efforts for rare conditions are challenging. Databases for rare diseases are not uniformly available, making both domestic and international epidemiological research complex. Attempts to understand patterns in rare diseases using large online search queries for case-report data have been successful to a degree for some regional studies [22], though data verification issues may continue to be problematic in this regard. Hence, the use of notifiable disease databases, such as the NNDSS, are increasingly important for providing data useful for epidemiological surveillance efforts [11].

The overarching aim of this study was to examine long-term longitudinal secondary data sets both to discern informative disease trends and to address the utility of such data to support surveillance efforts using verified databases as opposed to online search engines, as has been reported elsewhere [22]. One of the most notable characteristics of the results is the very large variability in the number of cases, and hence epidemiological rates, across the series. Though the cause of such heterogeneity has not been fully demonstrated, factors associated with changes in environmental conditions have been postulated. These include temporal environmental differences in weather/climate, changes in exposure parameters and outdoor activities, and variability in vector abundance and infectivity with pathogens [23]. Additional longer-term pattern variability with increasing cases since 2010 also includes the possibility of general increases in temperature through time, leading to increased environmental bacterial load, as well as the potential for increases in the number of tularemia genotypes and their exposure routes to humans, and enhanced ability to detect the pathogens using modern molecular techniques [4,23,24]. However, though these positive trends are evident since 2010 in reported cases to the CDC, there has not been such an increase in the inpatient records, which would presumably result from more severe cases of disease, perhaps suggesting that early interventional and/or prophylactic treatments after suspected exposure [25] or more rapid diagnosis using modern techniques [23] are preventing more outpatient cases converting to hospitalizations.

A postulation for the relatively large spike in NNDSS cases in 2015 could be related to changes in the transition from ICD-9 criteria to ICD-10 criteria, though if this were causative, a spike in NIS data might be expected as well, which was not the case. The drop in both reported cases and hospitalizations in 2020–2022 might be related to COVID-19 shutdowns, which might reduce environmental exposures and hence overall disease dynamics, as has been reported for other infectious diseases [26], in which case additional years of data, as they become available, would likely show a rebound of tularemia diagnoses.

Another hypothesis for changes in rates may be related to differences in *F. tularensis* subsp. *tularensis* (Type A), found in North America and largely associated with lagomorphs and associated tick species, versus *F. tularensis* subsp. *holarctica* (Type B), found in the northern hemisphere and found largely among rodents and aquatic species. Molecular evidence from the U.S. suggests that there are four distinct Type A variants (A1a, A1b, A2a, and A2b) along with Type B; further, differential disease etiology is apparent among the Type A variants, with the highest mortality seen in A1b [27]. There appears to be a distinct east–west variation as well, with A1 variants predominantly found in the central and eastern U.S. and A2 variants predominantly found in the western U.S. [27,28]. Consistent with the spatial distributions of cases shown in our analysis (Figure 5), Type A1a variants have been shown to predominate in the Midwest and West North Central regions of the U.S. from which the highest disease rates were also found; however, the NIS data indicated very few deaths across the study years, suggesting that prompt and adequate treatment is important in these regions. Unfortunately, however, *F. tularensis* subsp. typing is not differentiated in the secondary data sets available for our long-term temporal analysis, and hence differences in Type A/B pathology or case rates, per se, cannot be directly assessed. Hence, it is recommended that more granular analysis of laboratory-based testing data on tularemia be considered in future analysis.

One benefit of the using these databases as a pattern recognition exercise is that key characteristics among grouping variables can be seen. The NNDSS for reported cases across the time series, for example, suggests high case rates among White males with variable differences among ages, confirming previous reports [4,9]. Interestingly, this same pattern is true in the inpatient setting as well, confirming that certain patient characteristics are both at higher risk for infection and as a result are at higher risk for hospitalization. Strikingly, both NNDSS case rates and NIS hospitalization rates highlight the extraordinarily high risk among American Indian/Alaska Natives. The postulation is that this group may have greater environmental exposure geographically, with reservations in the central states with higher environmental loads and/or increased exposure to vectors or other infectious pathways resulting from occupational or culturally traditional practices (e.g., hunting and fishing) [4]. Detailed case discharge information from the NIS also suggests highly elevated rates of pulmonary tularemia among this group, supporting the increased risk among those working or hunting/trapping in high-risk geographies [29]. Further, increased risks for those working in outdoor environments, particularly landscaping jobs and construction work, employment categories currently dominated by male employees, may partially explain the high risk for disease and hospitalization among this group [30]. An important characteristic difference among American Indian/Alask Native populations, however, is that though case rates are uniformly highest among this group, hospitalizations are episodic (e.g., 2008, 2011, 2016, 2019, 2021, and 2022), highlighting once again the large variability in tularemia disease through time, with variable environmental conditions a likely candidate explanation for increased exposure probability [23], and documented deficiencies in access to healthcare a probable explanation for the lower numbers of hospitalizations in this group, a well-documented problem [31,32].

Unfortunately, with hospitalization comes the possibility of long stays and concomitant high costs, as the NIS data represent the most severe cases. One benefit of using inpatient discharge data for surveillance is understanding how hospitalization utilization rates change longitudinally and how these changes may be related to treatment costs [14,15]. With a median treatment cost of around USD 64,000 (in 2024 U.S. dollars) and a median length of stay of approximately a week, even rare diseases like tularemia can add significantly to the healthcare cost burden for patients and for hospitals. The importance of physician training to recognize early signs, and the appropriate use of antimicrobial treatment and prophylaxis in line with CDC recommendations, with the goal of reducing potential need of hospitalization except for only the most severe cases where intravenous antimicrobials are required, is important [25].

As stated in the introduction, an important underlying objective of the analysis was to investigate the utility of long-term secondary hospitalization data sets for indirect disease surveillance and their use as a potential surrogate for understanding patterns in diseases. Unfortunately, it does not appear that the last decade of tularemia data sufficiently captures the reported CDC case data. Though we were able to demonstrate that the expected case rate and or the expected hospitalization rates could be estimated using a simple linear regression, the effect size was small, and the ability to estimate such rates is likely due to data variability resulting in large overlaps in confidence intervals (Figure 1). Though it has been demonstrated that healthcare utilization records do provide useful information about costs for rare diseases [33], potential underestimation of case records and case rates using hospital records to project actual records may not provide the granularity necessary for precise estimation for the purposes of indirect epidemiological surveillance for this disease. However, this does not mean that understanding longitudinal patterns in hospitalization records is without value, as they do allow for unbiased estimates of tularemia types based on ICD diagnoses and for understanding useful patient characteristics that provide valuable insight into the types of patients, and hence the best treatment options, for those likely to be hospitalized for tularemia.

Analyses based on secondary data sets include several limitations, and we acknowledge these limitations herein. For the NNDSS data, extractions were based on annual reports and downloads from CDC databases in which historic records do not always differentiate between suspected/probable and confirmed cases and for which public-use data sets may not contain all available information; however, the overarching goal of this study was to investigate temporal period prevalence, in which total cases provide the bases for analysis. CDC summary reports [4,9] provide more granular details of probable and confirmed cases, and those reports are valuable complementary analyses to be examined in conjunction with this study, as some differences in analytical results can occur. Further, our aim was to utilize the CDC NNDSS data in conjunction with hospitalization data to provide context surrounding case rates, in which case we believe these limitations do not result in major analytical issues. Though the NIS data are known to be robust and are utilized in many studies, use of hospital discharge records comes with its own limitations and challenges. Kaulfus et al. provided an interesting summary of the challenges of using large, secondary data sets in health-based research [34]. They highlighted three main issues. The first issue highlighted problematic data extraction owing to the complexity of the data sets and the specialized analytical requirements necessary to use the data appropriately. Fortunately, we were able to directly address this limitation owing to our nearly decade-long experience working specifically with NIS data sets, and our ability to develop efficient extraction algorithms specifically for NIS data [15]. The second issue addressed sampling methodology because NIS utilizes a 20% weighted epidemiological sample that does not allow for analysis at the state level. Our intention was to provide summaries at the regional level in this study, and hence the spatial scale limitation was not inherently problematic. The third limitation concerned data storage and transmission; however, because we utilized a single repository for our data and centralized analyses among a local team, this was not an issue for our study. A final limitation of both the NNDSS and NIS data is that detailed, patient-level information supporting more granular analyses is not available in the secondary data. Hence, this limitation can only be addressed by finding new data sets, for example from electronic health records or laboratory data, from which additional variables can be analyzed. Regardless of these limitations, we contend that secondary data can provide a rich resource for indirect epidemiological surveillance while acknowledging the shortcomings of such data.

## Figures and Tables

**Table 1:** Diagnostic codes from the International Classification of Disease (ICD) used to extract tularemia cases.

Disease Type	ICD 9 (Ending 30 September 2015)	ICD 10 (Starting 1 October 2015)
Ulceroglandular tularemia	021.0	A21.0
Oculoglandular tularemia	021.3	A21.1
Pulmonary tularemia	021.2	A21.2
Gastrointestinal tularemia	021.1	A21.3
Generalized tularemia	021.8	A21.7
Other forms of tularemia	021.8	A21.8
Tularemia, unspecified	021.9	A21.9

**Table 2:** Incidence rates for tularemia, 2000–2022. Rates for the National Notifiable Disease Surveillance System (NNDSS) and the National Inpatient Sample (NIS) are provided based on categories in the existing databases. For NIS, International Classification of Disease (ICD) tularemia subcategory rates are reported where available.

Group/Variable	Total Incidence		NIS ICD Tularemia Type by Diagnostic Subcategory						
	NNDSS	NIS	Ulceroglandular	Oculoglandular	Pulmonary	Gastrointestinal	Generalized	Other forms	Unspecified
**Sex**									
Male	0.075	0.057	0.008	0.005	0.013	0.007	0.013	0.011	0.034
Female	0.039	0.029	0.006	0.002	0.005	0.003	0.008	0.008	0.018
**Age**									
<5	0.063	0.069	0.042	0.042	0.025	0.025	0.038	0.038	0.045
5–14	0.064	0.048	0.017	0.012	0.012	0.012	0.022	0.002	0.031
15–24	0.035	0.037	0.011	0.012	0.021	0.012	0.019	0.012	0.027
25–39	0.041	0.021	-	0.008	0.008	0.012	0.008	0.012	0.008
40–64	0.069	0.03	-	0.008	0.004	0.014	0.007	0.010	0.016
65+	0.080	0.067	0.010	0.009	0.014	0.018	0.017	0.016	0.043
**Ethnoracial Identity**									
White	0.053	0.042	0.007	0.003	0.007	0.004	0.010	0.008	0.024
Black or African American	0.011	0.026	0.011	0.012	0.016		0.012	0.024	0.018
Hispanic	0.014	0.026	0.008	-	0.011	0.008	0.009	0.017	0.018
Asian or Pacific Islander	0.015	0.027	-	-	-	-	-	-	0.027
American Indian or Alaska Native	0.508	0.178	-	-	0.183	-	-	-	0.176
Other or Multi-Race	0.073	0.116	-	-	0.069	-	0.047	0.047	0.100

- Indicates a cell in which data either are unavailable or are otherwise unreported owing to small cell count size (n < 10).

## Data Availability

All results reported were based on secondary data sets which can be obtained by interested parties through MOU with HCUP for NIS data [14] and through online tools from the CDC for NNDSS data [11].
